# Gut microbiome analysis of type 2 diabetic patients from the Chinese minority ethnic groups the Uygurs and Kazaks

**DOI:** 10.1371/journal.pone.0172774

**Published:** 2017-03-22

**Authors:** Ye Wang, Xin Luo, Xinmin Mao, Yicun Tao, Xinjian Ran, Haixia Zhao, Jianhui Xiong, Linlin Li

**Affiliations:** 1 Pharmacological Department, Basic Medicine College, Xinjiang Medical University, Urumqi, Xinjiang, China; 2 Pharmaceutical Preparation Department, the First Affiliated Hospital of Xinjiang Medical University, Urumqi, Xinjiang, China; University of Hawai'i at Manoa College of Tropical Agriculture and Human Resources, UNITED STATES

## Abstract

The gut microbiome may have an important influence on the development of diabetes mellitus type 2 (DM2). To better understand the DM2 pandemic in ethnic minority groups in China, we investigated and compared the composition and richness of the gut microbiota of healthy, normal glucose tolerant (NGT) individuals and DM2 patients from two ethnic minority groups in Xinjiang, northwest China, the Uygurs and Kazaks. The conserved V6 region of the 16S rRNA gene was amplified by PCR from the isolated DNA. The amplified DNA was sequenced and analyzed. An average of 4047 high quality reads of unique tag sequences were obtained from the 40 Uygurs and Kazaks. The 3 most dominant bacterial families among all participants, both healthy and DM2 patients, were the Ruminococcaceae, Lachnospiraceae, and Enterobacteriaceae. Significant differences in intestinal microbiota were found between the NGT individuals and DM2 patients, as well as between the two ethnic groups. Our findings shed new light on the gut microbiome in relation to DM2. The differentiated microbiota data may be used for potential biomarkers for DM2 diagnosis and prevention.

## Introduction

Diabetes mellitus (diabetes) is a metabolic disease, in which the patient has high blood sugar over a prolonged period of time. It is caused either by inadequate production of insulin, or the body's improper response to insulin, or both. It can cause many complications if left untreated, including cardiovascular disease, stroke, and kidney failure [1]. The International Diabetes Federation reports that the world has 415 million adults with diabetes and 318 million people at risk of developing diabetes [2].

There are three major types of diabetes: types 1 and 2, and gestational. Diabetes mellitus type 2 (DM2) accounts for 90% of all diabetes cases worldwide. It is closely related to unhealthy lifestyles, overweight and physical inactivity. Unhealthy diet, lack of exercise, and other unhealthy lifestyle habits are associated with the development of diabetes [3].

Xinjiang is an autonomous region of China, located in the northwest of the country. It is distinct for being the home of many ethnic groups including the Han, Uygurs, Kazaks, Hui, Kyrgyz, and Tajiks. Xinjiang consists mostly of mountains or deserts, and the prevalence of many diseases is unique to the area. For example, the prevalence of DM2 of the Uygurs (8.42%) is much higher than that of the Kazaks (1.56%) [4], while simultaneously the overall DM2 prevalence in China is about 1.7% [5].

Recent studies have suggested that the gut microbiome may be an important factor in the development of DM2 [6–7]. One study found that the guts of DM2 patients had a significantly lower proportion of the bacterial phylum Firmicutes and the class Clostridia [8]. To understand better the prevalence of DM2 in the Uygurs and Kazaks people of Xinjiang, and features associated with the gut microbiome in DM2, we investigated the gut microbiome of 40 Uygurs and Kazaks people, using the conserved V6 region of the bacterial 16S ribosomal DNA [9]. We compared the composition and richness (number of species) of the fecal microbial ecosystem of healthy and DM2 patients from both ethnic groups. To our best knowledge, this is the first study to compare the gut microbiome of DM2 patients from two unique ethnic groups in China. Our results provide new insight regarding the gut microbiome in relation to DM2. The distinct microbiota data may be used as potential biomarkers for DM2 diagnosis.

## Materials and methods

The Ethics Committee for Human Studies of First Affiliated Hospital of Xinjiang Medical University approved the protocol of this study (Approval number: 201402211–113, S1 Ethics Document). All participants provided written informed consent prior to sample collection.

### Human subjects

The study comprised 20 Uygurs and 20 Kazaks individuals, at least 3 generations of linear relatives. The Uygurs lived in HeTian, a town located in southwestern Xinjiang and populated almost exclusively by Uygurs. The Kazaks lived in Yili, a town in northwestern Xinjiang, where a large population of Kazaks lives. They were between 30 to 80 years old had lived in their respective towns for more than 20 years.

Women who were pregnant or lactating were excluded from this study. Also excluded were the patients with the following conditions: history of mental illness or drug abuse; surgery undergone or with other emergency situations; taking antibiotics, steroids or probiotics within the previous year; obvious liver and kidney dysfunction, gastrointestinal diseases, or serious diseases of the blood or the endocrine systems; severe heart diseases; systolic blood pressure > 160 mmHg and diastolic blood pressure > 95 mmHg; requiring insulin treatment or with a history of ketoacidosis; type I diabetics, gestational diabetics, or other particular types of diabetics.

The Uygurs and Kazaks groups were each stratified equally into those with DM2 (UDM2 and KDM2, respectively) and those with normal glucose tolerance (UNGT and KNGT), resulting in 4 groups of 10 persons each. DM2 was diagnosed according to the American Diabetes Association Guidelines for Diagnosis [10].

### Feces sample collection and DNA extraction

Feces samples were collected as described previously [7]. Briefly, stools were collected immediately after defecation and placed in the prepared centrifuge tube with culture medium. The tubes were placed on ice and sent to our laboratory. The samples were placed into plastic bags and stored at –80°C until processed further. DNA was extracted from approximately 0.18–0.2 g of stool sample, in accordance with the instructions of the kit (QIAamp DNA Stool Mini Kit, Qiagen, USA) [7]. The DNA concentration and quality was determined by a NanoDrop 1000 spectrophotometer (Thermo Scientific, USA).

### DNA sequencing of the 16S rRNA gene V6 region

The V6 region of the 16S rRNA gene was amplified by PCR from the isolated DNA. The PCR primers were: 967F, CAACGCGAAGAACCTTACC, and 1046R, CGACAGCCATGCANCACCT. The PCR conditions were: 94°C for 4 min, 94°C for 30 s; 55°C for 30s; 72°C for 10 s; 72°C for 5 min, 4°C for 30 cycles. The PCR products were purified and then sequenced using an Illumina HiSeq 2000 system (Illumina, USA). The Illumina sequencing strategy for the V6 region was paired-end and dual-end sequencing. The generated sequences were analyzed using Mothur software (http://www.mothur.org) for identification and annotation of unique target sequences (tags) and operational taxonomic units (OTUs).

### Tag and OTU analysis

The unique tag sequences were selected by redundancy removal using the Mothur software. They were then compared and aligned to the 16S rRNA gene V6 sequences in the nucleotide BLAST (BLASTN) database. The selected unique tag sequences were pre-clustered using the single linkage pre-clustering approach, with 98% similarity. Those 16S rRNA gene sequences whose similarity was >98% were defined as one OTU.

### Analysis of species richness and diversity

The dilution curve (rarefaction curve) was drawn to assess whether the amount of sequencing was sufficient to cover all groups, and to reflect the richness of species. The expected value of OTUs was calculated against the number of extracted tags (n value of tags) using measured relative proportions of OTUs of the 16S rRNA gene sequences. The rarefaction curve was then plotted by the expected value of OTUs versus n value of tags. When the curve reached its plateau, it was considered that the depth of sequencing had covered all species in the sample; i.e., that the sample had a higher species diversity, many of which have not been detected by sequencing. The horizontal axis represents the number of tags extracted (n value of tags); while the ordinate indicates the expected value of OTUs against the number of the extracted tags. Alpha diversity is the analysis of species diversity in a single sample, including Chao1, ACE values, and Shannon and Simpson indices. The Chao1 and ACE values were used to predict the type of microorganisms in the sample according to the measured quantity of tags and number of OTUs and their relative proportions. The Shannon is a diversity index, which combines the abundance and evenness of OTUs.

### Principal Coordinate Analysis (PCA) and Partial Least-Squares Discriminant Analysis (PLS-DA) for gut microbiota

The core bacteria of each group were obtained by the principal component analysis (PCA). The PLS-DA was then used to analyze the differences in intestinal flora between the NGT and DM2 groups.

### Cluster analysis

Cluster analysis was conducted using the R software package (V.2.9.1) (https://www.r-project.org/). Two kinds of *P*-value evaluation accuracy for clustering were provided: the approximately unbiased *P*-value and bootstrap probability (BP) *P*-value. The higher the *P*-value is, the higher the reliability.

### Statistical analysis

The abundance of the bacterial populations of the DM2 and NGT groups was assessed by the independent samples *t*-test using SPSS 19.0. The PCA and PLS-DA plots were generated using SIMCA software. The R software package was used for cluster analysis. *P* < 0.05 was considered statistically significant.

## Results

### Physical and biochemical indices of the Uygurs and Kazaks

No statistical differences were found regarding gender, or height between the DM2 and NGT individuals in either the Uygurs (Table 1) or Kazaks (Table 2) groups. However, significant differences were observed in many biochemical indicators, especially between the DM2 and NGT subjects in the Uygurs group. The differences between the NGT and DM2 individuals of the Kazaks population were less significant. The biochemical indicators also suggested that the Kazaks were healthier in general than the Uygurs. In addition, the ages between DM2 and NGT individuals in either the Uygurs or Kazaks were also significantly different, with the NGT groups younger than the DM2 groups (Tables 1 and 2). This may be a factor that affects the diversity of intestinal microbiota.

**Table 1:** Physical and biochemical indicators of NGT and DM2 groups in the Uygurs population.

	DM2	NGT
Subjects, n	10	10
Age, y	56.75 ± 16.26*	44.33 ± 15.61
Height, cm	162.25 ± 2.22	162.89 ± 7.29
Weight, kg	81.00 ± 5.83 *	71.49 ± 20.59
BMI, kg/m^2^	30.76 ± 1.94 *	28.23 ± 8.66
Waist, cm	111.33 ± 8.4 **	92.20 ± 18.63
FBG, mmol/L	11.49 ± 3.96 **	5.02 ± 0.77
TC, mmol/L	4.62 ± 0.60 *	3.92 ± 0.97
TG, mmol/L	1.68 ± 0.62 **	1.12 ± 0.48
HDL, mmol/L	1.11 ± 0.28 **	1.69 ± 0.98
LDL, mmol/L	3.64 ± 1.29	3.54 ± 0.79

* *P* < 0.05;

** *P* < 0.01, DM2 cf. NGT

**Table 2:** Physical examination and biochemical indicators of NGT and DM2 groups in Kazak population.

	DM2	NGT
Subjects, n	10	10
Age,y	48.33 ± 14.04*	35.29 ± 9.36
Height, cm	162.50 ± 4.28	162.33 ± 9.29
Weight, kg	60.40 ± 15.22	62.00 ± 19.10
BMI, kg/m^2^	22.57 ± 4.23	23.13 ± 4.55
Waist, cm	88.83 ± 16.24	81.29 ± 16.74
FBG, mmol/L	11.31 ± 1.77 **	5.10 ± 0.26
TC, mmol/L	4.15 ± 1.14	4.10 ± 0.72
TG, mmol/L	1.08 ± 0.50 **	0.43 ± 0.17
HDL, mmol/L	1.25 ± 0.74 *	3.81 ± 0.67
LDL, mmol/L	4.22 ± 2.13 *	2.82 ± 0.52

* *P* < 0.05;

** *P* < 0.01, DM2 cf. NGT

### Overall assessment of the intestinal microbiota of Uygurs and Kazaks

In total, we obtained 16.191 Gigabytes paired-end reads of the 16S rRNA gene V6 region from fecal samples of the 40 participants. After trimming and quality control analysis, an average of 4047 high quality reads was obtained (S1 Table). The KNGT group had the highest average number of reads (5491), while the UDM2 group had the lowest average reads (3232).

**Fig 1:**
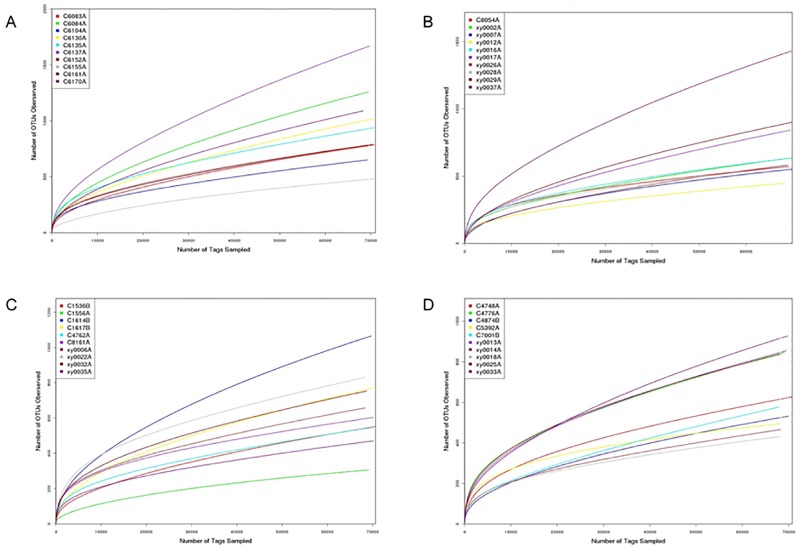
Rarefaction analysis of V6 sequencing tags of the 16S rRNA gene in fecal microbiota from adults with DM2 and NGT. (A) NGT group of Kazaks; (B) DM2 group of Kazaks; (C) NGT group of Uygurs; (D) DM2 group of Uygurs.

Each OTU is considered to represent a distinct microbial species. The number of OTUs also differed among the groups (S1 Table). The KNGT group had an average number of OTUs (946.4) that was higher than that of the KDM2 (716.5). However, the average OTUs of the UNGT (656.0) and UDM2 (659.8) groups were similar. The rarefaction curves in the 3% non-similarity level, which represents the species level, were generated (Fig 1).

Blast analysis of the 16S rRNA gene V6 region showed that more than 66% of tag sequences were aligned at the family level. Therefore, the fecal microbiotas of the 4 groups were further compared at the family level. Our results showed that the following three families were commonly shared among the four groups: Ruminococcaceae, Lachnospiraceae, and Enterobacteriaceae.

### Comparison of intestinal microbiota between the NGT and DM2 individuals of the Kazaks

**Fig 2:**
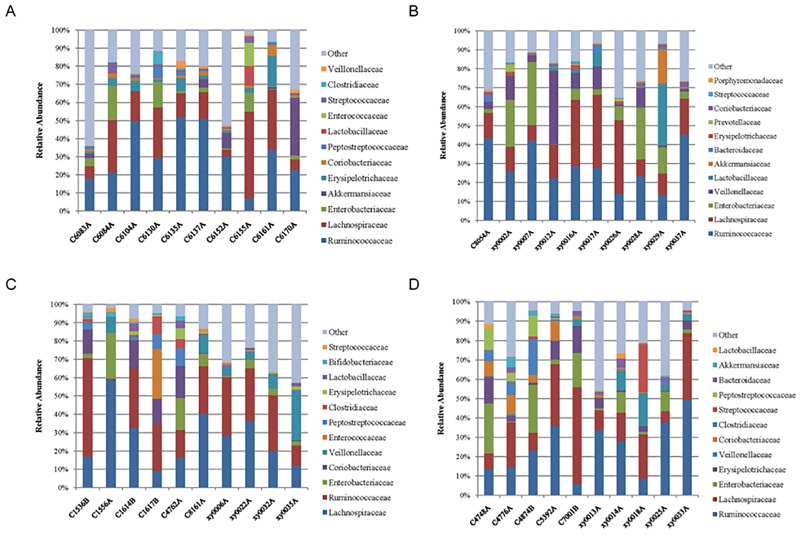
The tag numbers of the gut microbiota of each sample in the 4 groups at the family level. (A) NGT group of Kazaks; (B) DM2 group of Kazaks; (C) NGT group of Uygurs; (D) DM2 group of Uygurs.

**Fig 3:**
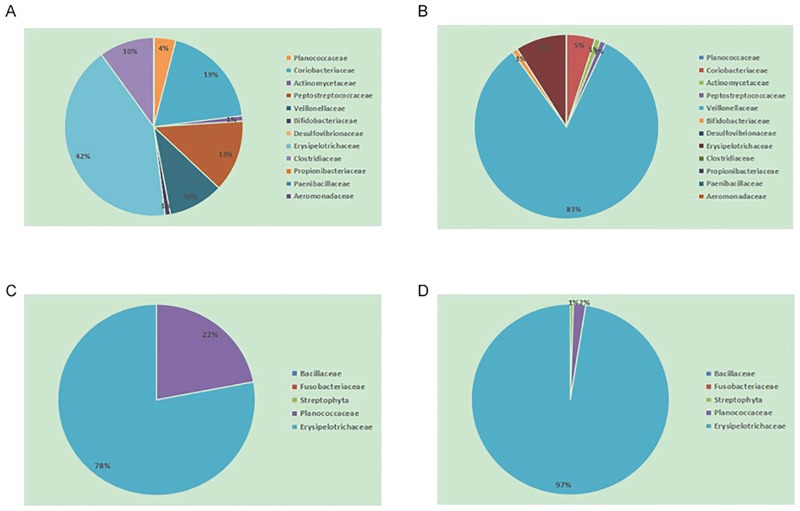
The fecal microbiota from NGT and DM2 individuals by profiling table. Data are represented as the average percentage of each profile. (A) NGT group of Kazaks, (B) DM2 group of Kazaks. The same color in (A) and (B) stands for the same gut flora in these two groups; (C) NGT group of Uygurs. (D) DM2 group of Uygurs. The same color in (C) and (D) stands for the same gut flora in these two groups.

**Fig 4:**
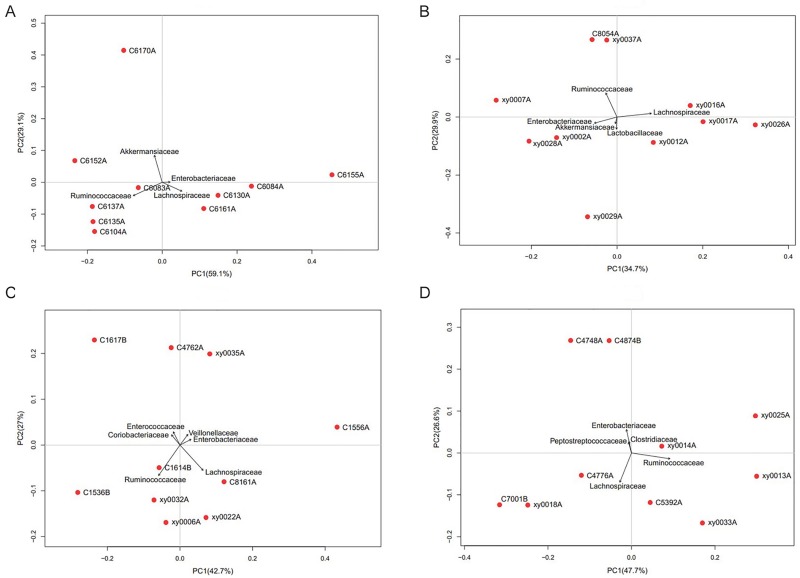
PCA analysis of the core gut microbiota of Uygurs and Kazaks. (A) NGT group of Kazaks (B) DM2 group of Kazaks (C) NGT group of Uygurs; (D) DM2 group of Uygurs.

**Fig 5:**
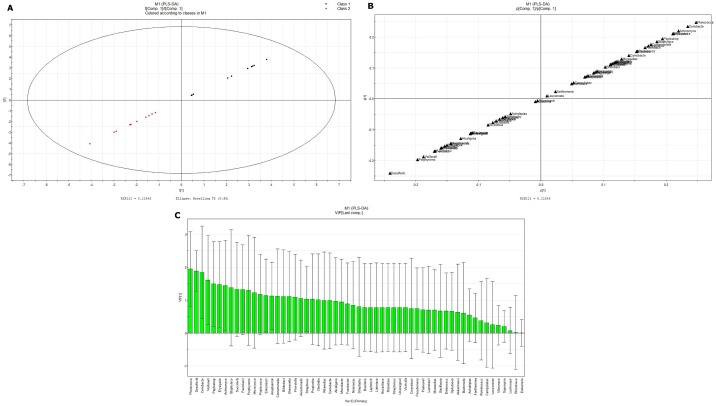
PLS-DA analysis of the overall difference between the intestinal flora of NGT and DM2 of Kazaks at the bacterial family level. (A) PLS-DA score-plot; (B) PLS-DA loading-plot; (C) VIP-plot.

Significant differences in microbial richness at the family level were found between the KNGT and KDM2 groups (Figs 2 and 3). For example, the Planococcaceae and Coriobacteriaceae families were highly enriched in the KNGT, while the Veillonellaceae was highly enriched in the KDM2 group (Table 3 and S2 Table). PCA analysis showed that the maximum variations were 59.1% (PC1) and 29.1% (PC2) in KNGT group, and 34.7% (PC1) and 29.9% (PC2) in the KDM2 group (Fig 4). PLS-DA analysis showed that the descending order for the contribution to the deviation of KNGT and KDM2 was: Planococcaceae, Desulfovibrionaceae, Coriobacteriaceae, Veillonellaceae, Erysipelotrichaceae, Actinomycetaceae, Staphylococcaceae, Succinivibrionaceae, Paenibacillaceae, Porphyromonadaceae, Mecorocoaceae, Petocoaceae, Enterobacteraceae, Anaplasmaaceae, Comamonaaceae, Bifidobacteraceae, Shewanelaceael, Prevotellacaceae, Aeromonaaceae, Streptococcaceae Propionibaceae, and Clostridiaceae (Fig 5).

**Table 3:** Differences of the gut microbial communities at family level between KNGT and KDM2 groups.

Family	KNGT, %	KDM2, %	*P* value
Planococcaceae	**0.41**	0.00	0.000
Coriobacteriaceae	**1.91**	0.51	0.001
Actinomycetaceae	**0.13**	0.001	0.002
Peptostreptococcaceae	**1.36**	0.08	0.002
Bifidobacteriaceae	**0.12**	0.002	0.005
Erysipelotrichaceae	**4.37**	0.99	0.011
Clostridiaceae	**1.02**	0.010	0.020
Propionibacteriaceae	**0.01**	0.00	0.020
Paenibacillaceae	**0.01**	0.00	0.029
Veillonellaceae	0.97	**9.29**	0.00
Desulfovibrionaceae	0.02	**0.12**	0.010
Aeromonadaceae	0.00	**0.01**	0.034

Numbers in bold letters indicate families that are enriched.

The KDM2 was predominantly linked to a higher score for Veillonellaceae, Desulfovibrionaceae, and Aeromonadaceae, while KNGT had a higher score for Planococcaceae, Coriobacteriaceae, Actinomycetaceae, Peptostreptococcaceae, Bifidobacteriaceae, Erysipelotrichaceae, Clostridiaceae, Propionibacteriaceae, and Paenibacillaceae. However, analysis of the species richness and diversity indices (Chao1, ACE, Shannon, Npshannon and Simpson) showed no significant differences between the KDM2 and KNGT groups (Table 4), suggesting that the differences were found mainly at the family level.

**Table 4:** Average number of OTUs and richness estimate indexes at 3% distance within fecal samples of the DM2 and NGT of Uygurs and Kazaks.

	KNGT	KDM2	UNGT	UDM2
OTUs	946.4 ± 336.89	716.5 ± 286.07	656.00 ± 211.49 *	659.80 ± 187.94 *
Chao1	2369.67 ± 992.13	1571.38 ± 774.07	1552.18 ± 527.26 *	1510.75 ± 500.40 *
ACE	4139.34 ± 2054.83	2602.07 ± 1625.33	2320.73 ± 766.38 *	2402.27 ± 930.98 *
Shannon	3.82 ± 0.57	3.54 ± 0.49	3.45 ± 0.70	3.68 ± 0.42
Npshannon	3.85 ± 0.58	3.56 ± 0.49	3.47 ± 0.71	3.70 ± 0.42
Simpson	0.06 ± 0.04	0.08 ± 0.05	0.09 ± 0.06	0.06 ± 0.02

* *P* < 0.05 (UNGT or UDM2 cf. KNGT)

### Comparison of intestinal microbiota between NGT and DM2 groups of Uygurs

No major differences in intestinal microbiota were found between the UNGT and UDM2 groups (Figs 2 and 3), except that the microbiota in the UDM2 group showed a significant decrease of Erysipelotrichaceae (5.56%) compared with the UNGT group (1.46%)(S3 Table). The average OTUs of the UNGT and UDM2 groups were slightly different but the difference was not statistically significant (*P* > 0.05; Table 4). The maximum variations by PCA were 42.7% (PC1) and 27% (PC2) in the UNGT, and 47.7% (PC1) and 26.6% (PC2) in UDM2 group (Fig 4).

### Comparison of intestinal microbiota between the Uygurs and Kazaks

Significant differences in intestinal microbiota were found between the Uygurs and Kazaks, especially between the KNGT group and the UNGT group, and between the KNGT and the UDM2 group. The average number of OTUs of the KNGT group was significantly higher than that of either the UNGT or the UDM2 groups (*P* < 0.05, Table 4). The variability of Chao1 and ACE estimates of the KNGT group were higher compared with that of the UNGT or UDM2 (Table 4). The UNGT group had a smaller percentage of the Planococcaceae family (KNGT = 0.41%, UNGT = 0.12%, *P* = 0.049) but a significant higher percentage of the Bacillaceae family (KNGT = 0.00%, UNGT = 0.02%, *P* = 0.014) and Streptophyta family (KNGT = 0.00%, UNGT = 0.02%, *P* = 0.037)(S4 Table).

**Fig 6:**
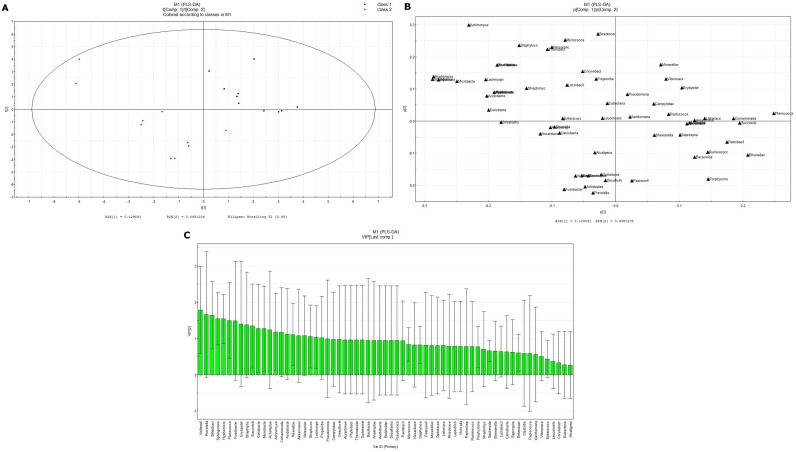
PLS-DA analysis of the overall difference between the intestinal flora of NGT of Kazaks and Uygurs at the bacterial family level. (A) PLS-DA score-plot; (B) PLS-DA loading-plot; (C) VIP-plot.

PLS-DA analysis showed a significant separation between the KNGT and UNGT groups at the level of bacterial family, suggesting a significantly different composition of microbiota between these two groups. According to the variable influence on the projection (VIP) values, the descending order for the contribution to the deviation of KNGT and UNGT was: Veillonellaceae, Prevotellaceae, Bifidobacteraceae, Sphingomonaceae, Hyphonicrobiaceae, Planococcaceae, Fusobacteriaceae, Erysipelotrichaceae, Streptophytaaceae, Succinivibaceae, Croiobacteraceae, Microbacteriaceae, Acholeplaaceae, Actinomyaceae, Comamonadaceae, Acidobacteraceae, Rikenellaceae, Akkermansiaceae, Neisseriaceaceae, Sterptococcaceae Lachnospiraceae and Propionibacteraceae (Fig 6). The KNGT group was predominantly linked to a higher score for Bacillaceae, Fusobacteriaceae and Streptophyta, while the UNGT groups had a higher score on Planococcaceae.

**Fig 7:**
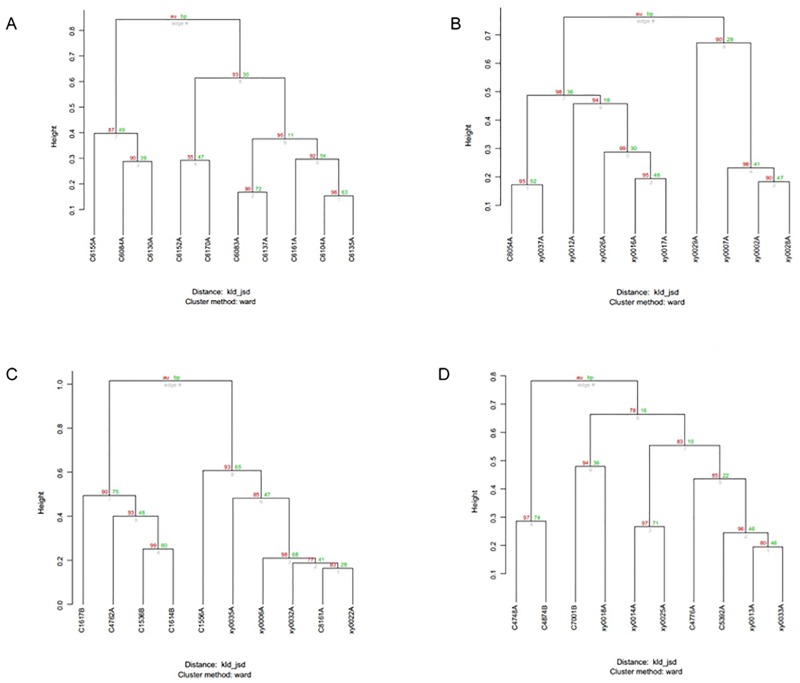
Clustering analysis of each group of Kazaks and Uygurs at the bacterial family level. A) NGT group of Kazaks; (B) DM2 group of Kazaks; (C) NGT group of Uygurs; (D) DM2 group of Uygurs.

**Fig 8:**
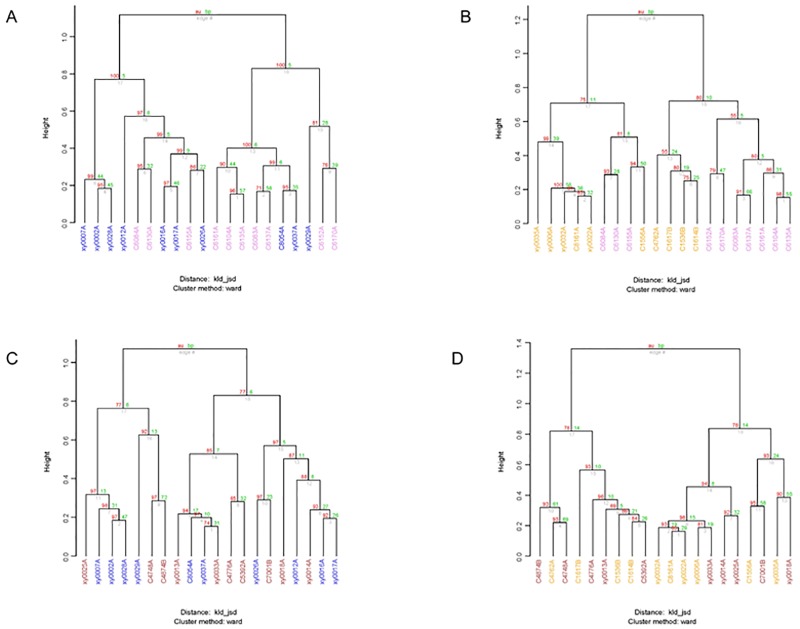
Clustering analysis of the NGT and DM2 groups of Kazaks and Uygurs at the bacterial family level. (A) NGT group of Kazaks; (B) DM2 group of Kazaks; (C) NGT group of Uygurs; (D) DM2 group of Uygurs. Two *P*-values were provided: AU (Approximately Unbiased) *P*-value (red), and BP (Bootstrap Probability) *P*-value (green).

Clustering at the family level was observed among individual samples in each group (Figs 7 and 8). The KNGT group is gathered more obviously than the KDM2 group, and the UNGT or UDM2 groups. This suggests that the KNGT group has higher microbial similarity than the other three groups. Cluster analysis of the different groups showed that the NGT of Kazaks and Uygurs were clustered (Fig 8), further suggesting that the intestinal microflora composition of these two groups is different. In addition, the NGT and DM2 of Kazaks were also clustered.

## Discussion

In this study, we report a comparison of the gut microbiota of two ethnic groups, the Uygurs and Kazaks, in the northwest and southwest region of Xinjiang in China. These two groups live mostly in mountains and deserts with diets that are unique to their ethnic groups. In a 2005–2007 survey, the Uygurs showed a much higher prevalence of DM2 than the Kazaks [4].

Our data indicated that the Ruminococcaceae, Lachnospiraceae, and Enterobacteriaceae are the three dominant bacterial families in both healthy and DM2 patients of the Kazaks and Uygurs. The Ruminococcaceae and Lachnospiraceae are commonly found in intestinal microbiota and their proportion can be altered by high-calorie diets [11], a possible cause of DM2. The Enterobacteriaceae is a family of Gram-negative bacteria that includes many pathogens (such as *Salmonella* and *Escherichia coli*), as well as many harmless symbionts. *Aeromonas* species, for example, are members of the Enterobacteriaceae family and exhibit oxidase activity and glucose fermentation [12]. They have been reported in association with chronic diseases, such as diabetes [13]. A further detailed dissection of this family would greatly benefit our understanding of the possible involvement of the large family of Enterobacteriaceae in DM2.

Our results showed a higher level of Desulfovibrionaceae and Aeromonadaceae in DM2 patients of Kazaks. This is consistent with a previous report [7], which shows a significant enrichment of the Proteobacteria, a class to which Desulfovibrionaceae and Aeromonadaceae belong. Desulfovibrionaceae is a strict anaerobic and sulfate-reducing bacterial family [8]. An earlier report showed that *Desulfovibrio* sp. were enriched in DM2 patients [14]. A higher positive score for Veillonellaceae was also found in KDM2 patients. Veillonella is a non-fermentative, strictly anaerobic, and gram-negative coccus that is part of the human gastrointestinal tract, mouth and vaginal flora. It is considered a pathogen in diabetic patients with osteomyelitis and those with underlying immune suppression [15].

The DM2 patients of the Uygurs also showed a higher level of Erysipelotrichaceae, which has also been reported with a higher level in mice on a high fat or Western diet [16]. There is also strong evidence of a correlation between levels of Erysipelotrichaceae and host cholesterol metabolites [17], or inflammation [18, 19]. This correlation may eventually be used as a promising microbial target to combat metabolic disorders. In our current study, we found that the level of Erysipelotrichaceae in the UDM2 group (5.56%) is much higher than that in the UNGT (1.46%), while majority of the intestinal microbiota were similar between these two groups. It suggests that Erysipelotrichaceae might be the only major indicator that differentiates the UDM2 from the UNGT. However, the level of Erysipelotrichaceae was higher in the KNGT (4.37%) group than in the KDM2 (0.99%) group, suggesting that high levels of Erysipelotrichaceae may be a unique feature of DM2 patients of the Uygurs. It also suggests that the level of Erysipelotrichaceae in Kazaks may have an opposite correlation in terms of metabolic disorders. One limitation of this study is that the ages between the DM2 and NGT groups of either Uygurs or Kazaks are different, which may affect both the composition and level of intestinal microbiota.

Diabetes not only affects the abundance of certain bacterial families or species of gut microbiota, but also the composition [20, 21]. In this study, we found that the KNGT group had a higher average number of OTUs than did the KDM2. The cluster analysis suggested that the intestinal microflora composition between these two groups was different. Less significant difference was found between the UKNG and UDM2 groups, suggesting that intestinal microflora composition varies among different ethnic groups. In addition, we found a significant difference in intestinal microbiota between the Uygurs and Kazaks, especially between the KNGT group and the UNGT group and between the KNGT and the UDM2. The average number of OTUs of the KNGT was significantly higher than that of either the UNGT or the UDM2 groups. One possible reason for this difference is the differences of their geographic locations. The Uygurs live in southwestern Xinjiang, where it has a cold desert climate, with only about 36.5 mm of annual precipitation. The Kazaks, on the other hand, live in northeastern Xinjiang. It has a colder, semi-desert climate, but with a much larger precipitation (~ 300 mm annually). Considering that the Kazaks have a much lower rate of DM2, this results suggest that the higher number of microbes in the Kazaks of normal glucose tolerance may be beneficial.

More research is underway to determine the possible beneficial microbes in the Kazaks ethnic group that could potentially be used to prevent or reduce DM2 in a larger community.

In conclusion, our study revealed a significant difference in gut microbiota between DM2 and healthy individuals, as well as between different ethnic groups in northwest China. The findings shed new light on a possible association between gut microbiota and diabetes prevalence. The microbiota data may also be used for as potential biomarkers for the DM2 diagnosis and prevention.

## Supporting information

S1 Ethics Document (TIFF)

S1 Table The sequence analysis of intestinal microbiota from the 40 samples. (TIFF)

S2 Table Significant average abundance difference between KNGT and KT2DM at the family level. (TIFF)

S3 Table Significant average abundance difference between KNGT and UNGT at the family level. (TIF)

S4 Table Significant average abundance difference between UNGT and UDM2 at the family level. (TIF)
