# Correction: Gut microbiome analysis of type 2 diabetic patients from the Chinese minority ethnic groups the Uygurs and Kazaks

**DOI:** 10.1371/journal.pone.0249015

**Published:** 2021-03-24

**Authors:** Ye Wang, Xin Luo, Xinmin Mao, Yicun Tao, Xinjian Ran, Haixia Zhao, Jianhui Xiong, Linlin Li

Following publication of this article [1], concerns were raised about the availability of the underlying data. This notice updates readers about the availability of the data with a corrected Data Availability statement.

The Data Availability statement is as follows:

The sequence and assembly data for each of the 40 gut microbiota samples is deposited at the CNGB Sequence Archive (https://db.cngb.org/cnsa/) under project accession number CNP0001174. The associated individual-level demographic data is deposited under accession number CNP0001169 (restricted data, for access requests visit https://db.cngb.org/data_access/).

Sample accession numbers are provided as Supporting Information on this notice (S1 File). Samples are grouped as follows:

Kazak, normal glucose tolerance (KNGT) = samples 1–10 (sample accessions CNS0254252-CNS0254261)

Kazak, diabetes mellitus type 2 (KDM2) = samples 11–20 (sample accessions CNS0254262—CNS0254271)

Uygur, normal glucose tolerance (UNGT) = samples 21–30 (sample accessions CNS0254272—CNS0254281)

Uygur, diabetes mellitus type 2 (UDM2) = samples 31–40 (sample accessions CNS0254282—CNS0254291)

Previously published studies on the same samples used 16S rDNA real time PCR to assess correlation between genus-level abundance of bacteria with physical and biochemical indicators in the same four groups [2]. Another previous study on the same samples reported physical and biochemical measurements as well as levels of indirect markers of bacterial exposure in the same four groups [3].

The following additional clarifications are provided regarding statistical analyses in [1]:

Sample size calculation

The sample size calculation uses the following experimental data from [2]:

By 16S rDNA real time PCR in genus level, the abundance of Veillonella spp and C. coccoides subgroups in Kazaks was obtained (KNGT VS KDM2: 2.10±0.20 VS 3.05±0.39; KNGT VS KDM2: 4.27±0.32 VS 4.80±0.19 Fig 1 and 2 of [2]). According to the following formula, we calculated the sample sizes of Kazaks as 4.47and 10.35, respectively.

The sample size calculation uses the following formula to compare two sample means:

$$n=\frac{{\left(Z_{\alpha }+Z_{\beta }\right)}^{2}*2\sigma^{2}}{\delta^{2}}$$

Where numerator: σ is total variance; denominator: δ is mean difference between two groups. α = 0.05, β = 0.9, Z_α_ = 1.96 and Z_β_ = 1.28

Considering the previously published prevalence of KDM2 (1.47%) [4] and the difficulties collecting KDM2 samples, the number of feces samples for Illumina sequencing was 10 (shown in table 1 and 2 [1]).

Data in Tables 1, 2 and 4 of [1] are reported as mean ± SD.

## Supporting information

S1 File Sequence data accession numbers. (DOCX)

## References

[1] Wang Y, Luo X, Mao X, Tao Y, Ran X, Zhao H, et al. (2017) Gut microbiome analysis of type 2 diabetic patients from the Chinese minority ethnic groups the Uygurs and Kazaks. PLoS ONE 12(3): e0172774. 10.1371/journal.pone.0172774 28328990 PMC5362050

[2] Zhao H, Li L, Mao X, Zhang R, Xiong J, Wang Y. The relationship of Veillonella spp and C. coccoides subgroup levels in gut microbiota with type 2 diabetes mellitus in Uygurs and Kazaks of Xinjiang. Chinese Journal of Microecology. 2015;27(1):6–10. 10.13381/j.cnki.cjm.201501002

[3] Zhang P, Li L, Xiong J, Zhao H, Mao X, Wang Y. The relationship of LBP, sCD14 levels with type 2 diabetes mellitus in Uygurs and Kazaks of Xinjiang. Chinese Journal of Microecology. 2014;26 (2):139–143. 10.13381/j.cnki.cjm.201402004

[4] Tao Y, Mao X, Xie Z, Ran X, Liu X, Wang Y, et al. The Prevalence of Type 2 Diabetes and Hypertension in Uygur and Kazak Populations. Cardiovasc Toxicol. 2008; 8:155–159. 10.1007/s12012-008-9024-0 18777166

