# Supplementary figures and images for: Gut microbiome analysis of type 2 diabetic patients from the Chinese minority ethnic groups the Uygurs and Kazaks
Source: PLoS One. 2017 Mar 22;12(3):e0172774. doi: 10.1371/journal.pone.0172774 (PMC5362050; doi:10.1371/journal.pone.0172774)

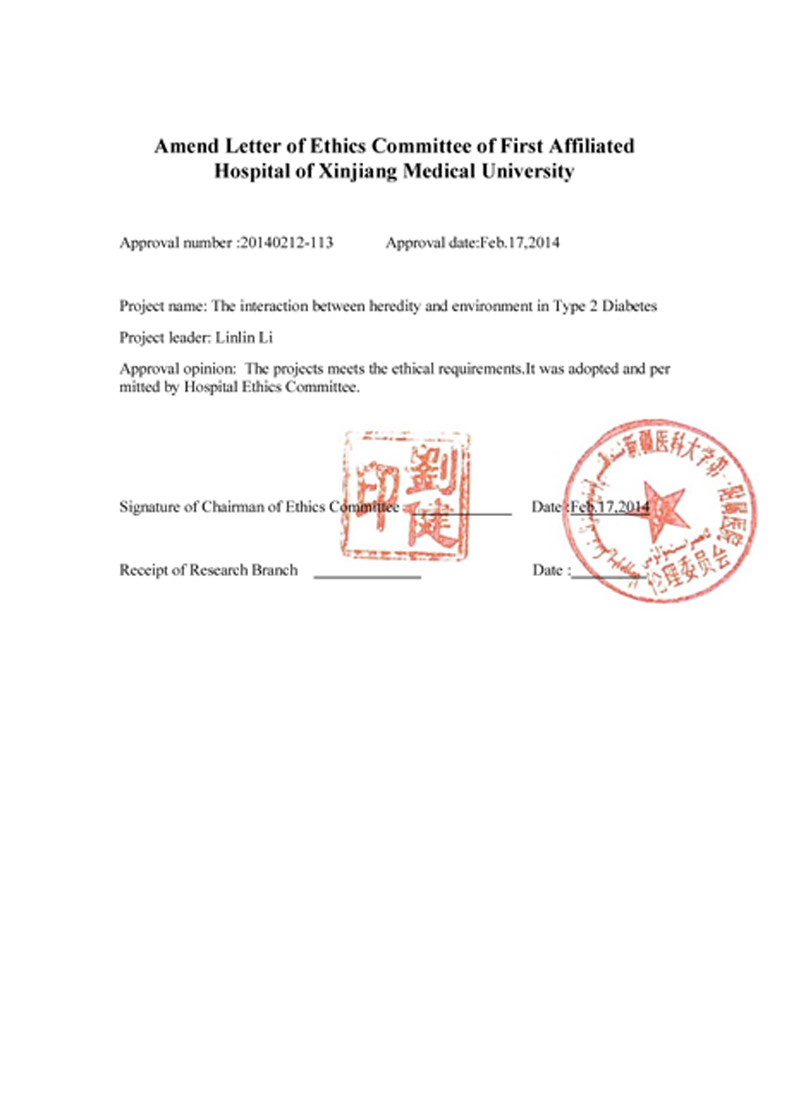

Supplement: S1 Ethics Document — (TIFF) [file pone.0172774.s001.tiff]
