# Supplementary material for: Correction: Gut microbiome analysis of type 2 diabetic patients from the Chinese minority ethnic groups the Uygurs and Kazaks
Source: PLoS One. 2021 Mar 24;16(3):e0249015. doi: 10.1371/journal.pone.0249015 (PMC7990291; doi:10.1371/journal.pone.0249015)
Supplement: S1 File — (DOCX) [file pone.0249015.s001.docx]

| Sample | Project accession | Sample accession | Assembly accession |
| --- | --- | --- | --- |
| 1 | CNP0001174 | CNS0254252 | CNA0014591 |
| 2 | CNP0001174 | CNS0254253 | CNA0014592 |
| 3 | CNP0001174 | CNS0254254 | CNA0014593 |
| 4 | CNP0001174 | CNS0254255 | CNA0014594 |
| 5 | CNP0001174 | CNS0254256 | CNA0014595 |
| 6 | CNP0001174 | CNS0254257 | CNA0014596 |
| 7 | CNP0001174 | CNS0254258 | CNA0014597 |
| 8 | CNP0001174 | CNS0254259 | CNA0014598 |
| 9 | CNP0001174 | CNS0254260 | CNA0014599 |
| 10 | CNP0001174 | CNS0254261 | CNA0014600 |
| 11 | CNP0001174 | CNS0254262 | CNA0014601 |
| 12 | CNP0001174 | CNS0254263 | CNA0014602 |
| 13 | CNP0001174 | CNS0254264 | CNA0014603 |
| 14 | CNP0001174 | CNS0254265 | CNA0014604 |
| 15 | CNP0001174 | CNS0254266 | CNA0014605 |
| 16 | CNP0001174 | CNS0254267 | CNA0014606 |
| 17 | CNP0001174 | CNS0254268 | CNA0014607 |
| 18 | CNP0001174 | CNS0254269 | CNA0014608 |
| 19 | CNP0001174 | CNS0254270 | CNA0014609 |
| 20 | CNP0001174 | CNS0254271 | CNA0014610 |
| 21 | CNP0001174 | CNS0254272 | CNA0014611 |
| 22 | CNP0001174 | CNS0254273 | CNA0014612 |
| 23 | CNP0001174 | CNS0254274 | CNA0014613 |
| 24 | CNP0001174 | CNS0254275 | CNA0014614 |
| 25 | CNP0001174 | CNS0254276 | CNA0014615 |
| 26 | CNP0001174 | CNS0254277 | CNA0014616 |
| 27 | CNP0001174 | CNS0254278 | CNA0014617 |
| 28 | CNP0001174 | CNS0254279 | CNA0014618 |
| 29 | CNP0001174 | CNS0254280 | CNA0014619 |
| 30 | CNP0001174 | CNS0254281 | CNA0014620 |
| 31 | CNP0001174 | CNS0254282 | CNA0014621 |
| 32 | CNP0001174 | CNS0254283 | CNA0014622 |
| 33 | CNP0001174 | CNS0254284 | CNA0014623 |
| 34 | CNP0001174 | CNS0254285 | CNA0014624 |
| 35 | CNP0001174 | CNS0254286 | CNA0014625 |
| 36 | CNP0001174 | CNS0254287 | CNA0014626 |
| 37 | CNP0001174 | CNS0254288 | CNA0014627 |
| 38 | CNP0001174 | CNS0254289 | CNA0014628 |
| 39 | CNP0001174 | CNS0254290 | CNA0014629 |
| 40 | CNP0001174 | CNS0254291 | CNA0014630 |

[Table](http://www.ncbi.nlm.nih.gov/pmc/articles/PMC2816710/table/pone-0009085-t002/) **1.** The sample and assembly accession
